# Atraumatic Flexor tendon retrieval- a simple method

**DOI:** 10.1186/1750-1164-7-11

**Published:** 2013-09-16

**Authors:** Muhammed Besir Ozturk, Salih Onur Basat, Turgut Kayadibi, Mehmet Karahangil, İsmail Mithat Akan

**Affiliations:** 1 Department of Plastic Reconstructive and Aesthetic Surgery, Okmeydani Training and Research Hospital, İstanbul, Turkey; 2 Department of Plastic Reconstructive and Aesthetic Surgery, Medipol University Hospital, İstanbul, Turkey; 3 Şahin sokak Karaşahin Apt No: 10/9, Beyoğlu/Istanbul 34445, Turkey

**Keywords:** Flexor tendon retrieval, Atraumatic flexor tendon retrieval, Tendon retrieval, Retracted flexor tendon

## Abstract

**Background:**

Zone 2 flexor tendon injuries still represent a challenging problem to hand surgeons despite the well developed surgical techniques and suture materials. Meticulous surgical repair with atraumatic handling of the severed tendon stumps and minimal damage to the tendon sheath are particularly important to prevent postoperative adhesions and ruptures in this area.

In zone 2 flexor tendon injuries proximal to the vinculas, the cut ends of the flexor tendons retract to the palm with muscle contraction. To retrieve the severed proximal flexor tendon under tendon sheath and pulley system is very difficult without damaging these structures. Many techniques are described in the literature for the delivery of the retracted proximal tendon stump to the repair site.

**Methods:**

In this report we would like to present a simple and relatively atraumatic technique that facilitates passing of the retracted flexor tendon through the pulleys in zone 2. We sutured the proximal tendon stump at the distal palmar crease with 3–0 polypropylene suture and used a 14 gauge plastic feeding tube, acting like a conduit for the passage of straightened needle to the finger.

**Results:**

We have used this technique 21 times without any complication in our clinic. We have not seen any suture breakage during the passage or needle breakage due to the bending of the needle.

**Conclusions:**

We have found this technique is very simple and very effective in retrieving the retracted tendon stump without causing undue damage to the tendon stump or tendon sheath.

## Background

Zone 2 flexor tendon injuries still represent a challenging problem to hand surgeons despite the well developed surgical techniques and suture materials. Meticulous surgical repair with atraumatic handling of the severed tendon stumps and minimal damage to the tendon sheath are particularly important to prevent postoperative adhesions and ruptures in this area.

In zone 2 flexor tendon injuries proximal to the vinculas, the cut ends of the flexor tendons retract to the palm with muscle contraction. To retrieve the severed proximal flexor tendon under tendon sheath and pulley system is very difficult without damaging these structures.

Various techniques have been described in the literature for the delivery of the retracted proximal tendon stump to the repair site, including the milking of the proximal tendon stump [1], suction [2], use of rigid and flexible tendon retrievers [3], skin hooks [4], steel wires [5], aneurismal needle [6], tendon suture side to side to a catheter [7] and endoscopic tendon retrieval [8].

In this report we would like to present a simple and relatively atraumatic technique that facilitates passing of the retracted flexor tendon through the pulleys in zone 2.

## Methods

**Figure 1:**
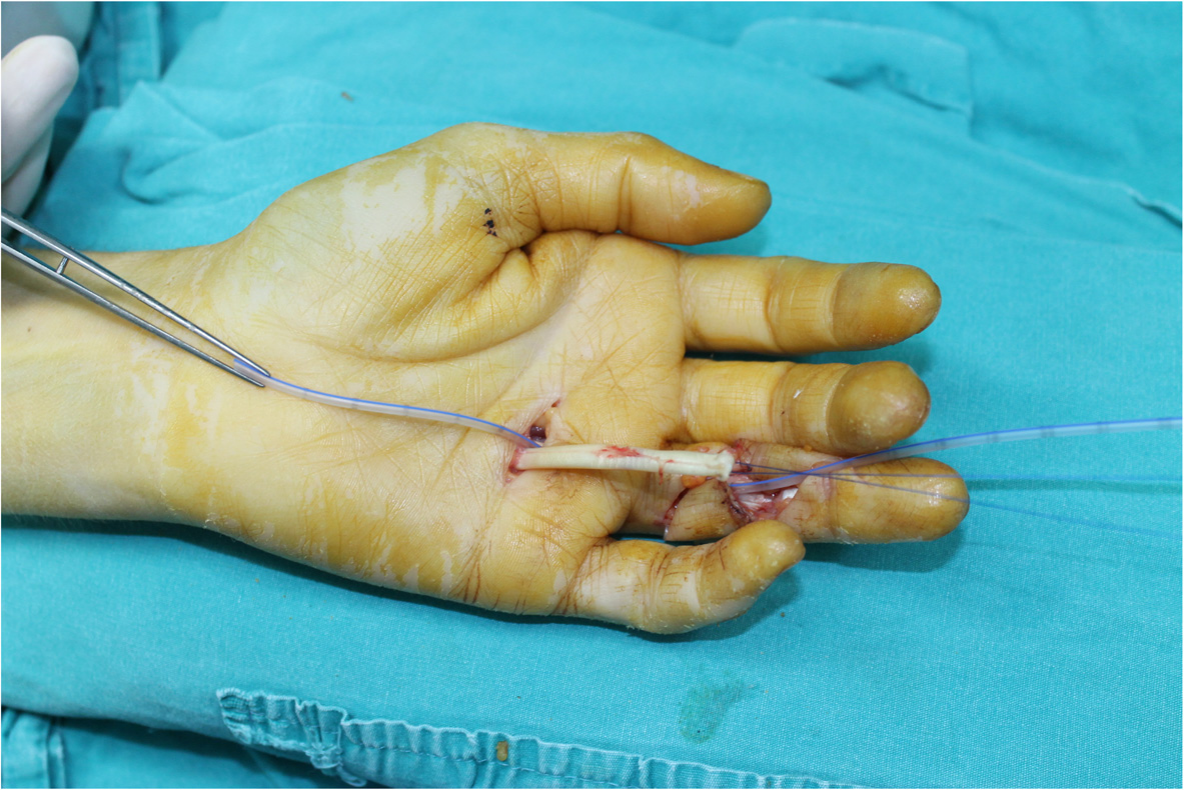
**The proximal end of the retracted tendon is delivered outside the skin.** The suction catheter is passed through the tendon sheath and it is ready to be used as a tunnel under the pulley system. The permanent half modified Kessler suture is placed in the proximal tendon end with 3–0 polypropylene suture.

**Figure 2:**
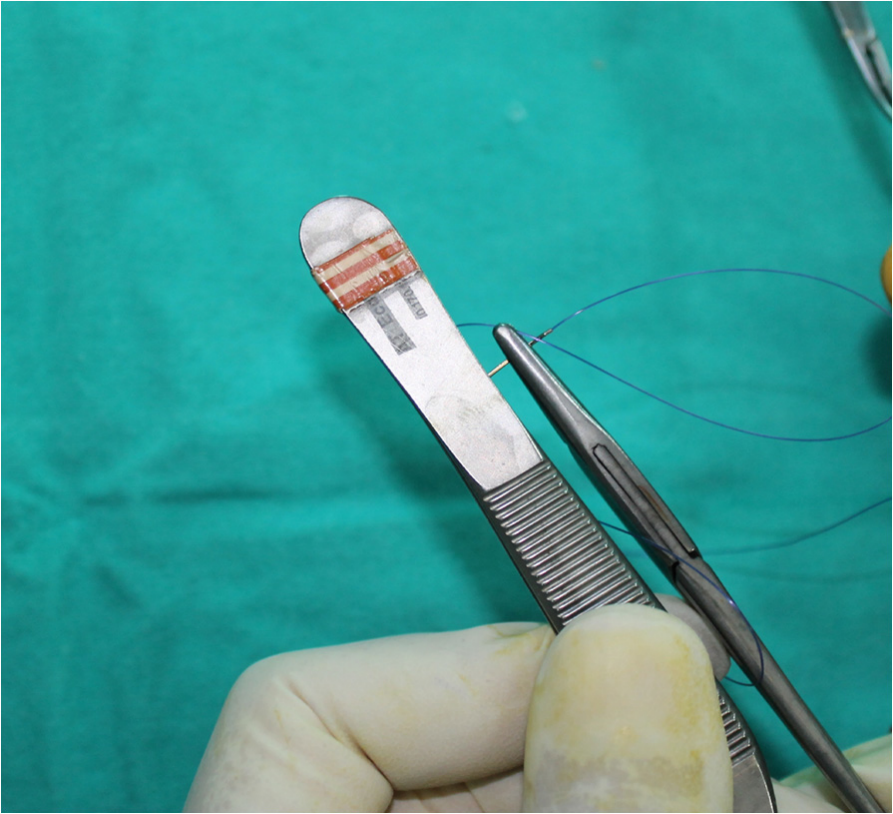
The curved suture needle is straightened with fine forceps.

**Figure 3:**
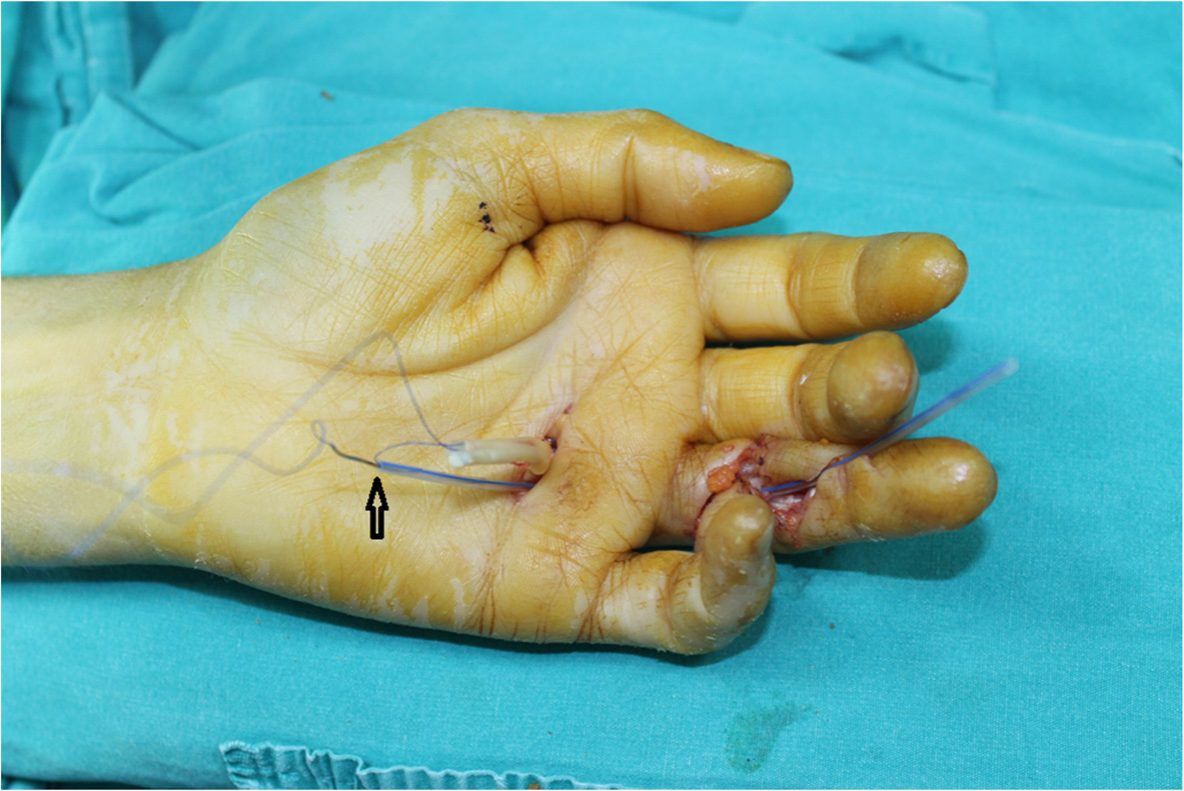
**The straightened needle and the distal end of the suture are threaded through the catheter.** Arrow indicates the insertion of the straightened needle into the catheter.

The primary wound is expanded proximally and distally by standard Brunner incisions and the cut tendon stumps are explored. If we fail to find the proximal tendon stump at the site of injury another incision is made in the distal palmar crease. The flexor tendon sheath proximal to the A1 pulley is opened by a transvers incision. The retracted tendon is exposed and it is delivered outside the skin. Next bullet end of an 8-French (F) suction catheter is passed through the opened proximal tendon sheath distally until it exits through the distal tendon sheath at the repair place. The permanent half modified Kessler suture is placed in it with 3–0 polypropylene suture (Figure 1). The curved suture needle is straightened with fine forceps (Figure 2). The bullet end is cut so the catheter is ready to be used as a tunnel under the tendon sheath. The straightened needle and the distal end of the suture are threaded through the catheter by hand (Figure 3). The distal end of suture should be threaded through the catheter till it exits the distal end of it. The insertion of the needle into the catheter is enough and the complete passage of the needle through the catheter is not necessary. The full insertion of the proximal end of the needle into the catheter is important to prevent the damage to the tendon sheath during the passage. The length of the sutures should be adjusted well so that once the catheter is taken out from the finger, the needle and the distal end of the suture should be reach to the repair place.

**Figure 4:**
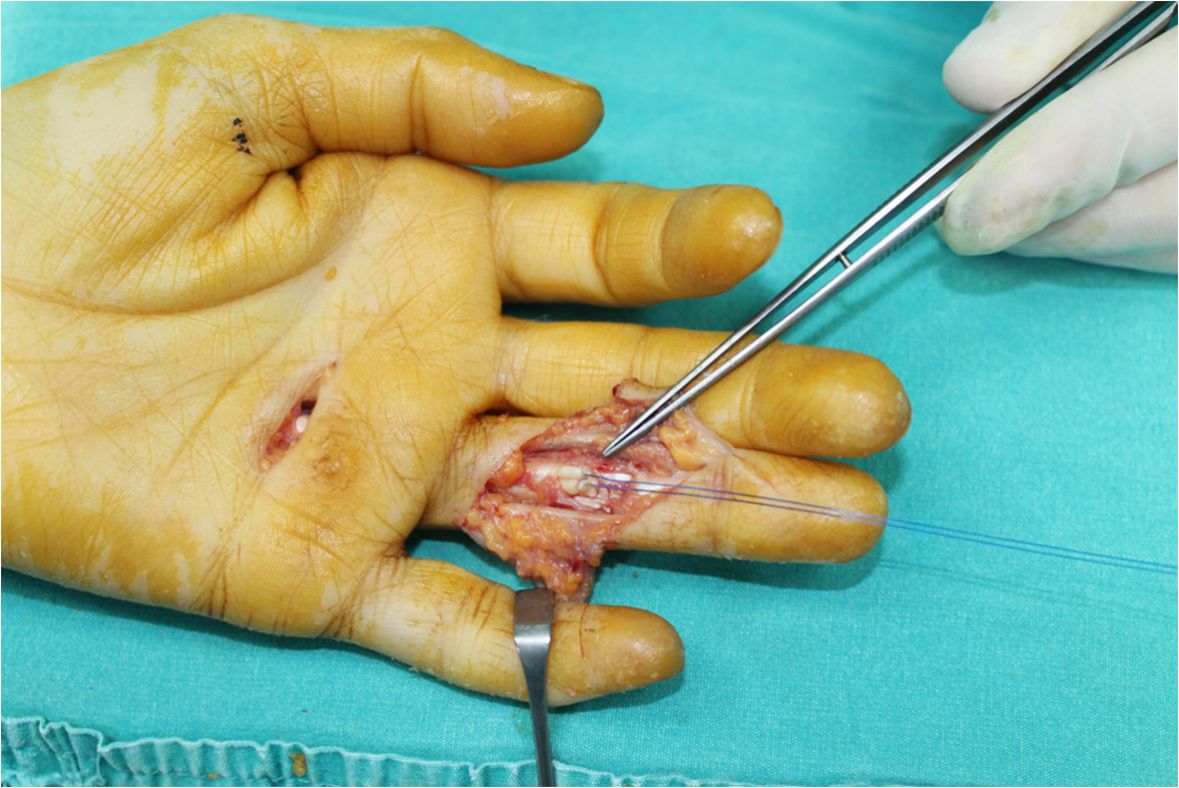
**The catheter is taken out from the finger leaving the needle and the distal end of the suture at the repair place.** With continuous gentle traction proximal tendon stump is delivered to the distal site and it is ready to be repaired.

With gentle traction on suture ends and with the help of a fine forceps, the cut end of the proximal tendon is inserted in the tendon sheath at the distal palmar crease. With continuous gentle traction, the tendon is delivered into the finger (Figure 4). The proximal tendon is fixed in place by 25-gauge needle. Distal stump is usually identified by flexing the distal interphalangeal joint. The straightened needle is curved again and the other half of the Kessler suture is completed. The epitendinous suture is then completed with 6.0 polypropylene suture.

If the proximal tendon stump further retracts into the palm and we fail to find it at the distal palmar crease, a third incision is made in the wrist crease. The retracted tendon is explored and once it is found, it is delivered outside the skin at the wrist. We have found it difficult to bring the tendon stump from wrist to the repair site directly, because of the excessive tension on the suture. So, using the same technique as described above, the proximal tendon stump is first retrieved from the wrist to the distal palmar crease. It is then retrieved from distal palmar crease to the repair place.

## Results and discussion

Flexor tendon repair in zone 2 requires an atraumatic tendon retrieval technique with meticulous handling of the tendon stumps and minimal damage to the tendon sheath because of the problematic tendon healing nature of this zone.

There are various techniques for flexor tendon retrieval beneath the pulleys. Rigid and flexible tendon retrievers create additional crush injury to the severed tendon stump which causes additional fibrosis and altered tendon healing [4,5]. The suction catheter we use is very inert and only needs to be passed once through the sheath. It has a smooth surface and its bullet end does not damage the tendon sheath.

Hettiaratchy and Titley described placing a core suture in the tendon stump and using an aneurysm needle to thread these sutures through the pulleys and pull the tendon out with this sutures [7]. This technique is not suitable to use for tendons retracted to palm and requires additional windowing of the sheath which means additional damage. The suction catheter we use is readily available in the operating room and it is long enough to reach the distal palmar crease.

Other methods, passing a silastic tube through the tendon sheath and then suturing the proximal tendon stump to the tube side to side, end to end or placing the tendon within its lumen have been described [7,9,10]. But all these methods create a bulky mass which may harm the tendon sheath during the passage. Also all these methods require the tendon stumps be sutured multiple times which may fray the tendon tip and make the repair more difficult. In our technique there are no additional sutures to tenorraphy sutures and this minimizes tendon trauma. The use of only polypropylene sutures to retrieve the proximal tendon stump minimizes the thickness and facilities the passage.

Bhatti described a technique similar to us, suturing the proximal tendon stump at the distal palmar crease with straight needle polypropylene suture and using 14 gauge plastic cannula with stylet, acting like a conduit for the passage of straight needle to the finger [11]. Traction of the suture end results in delivery of cut tendon stump. The straight needle is not always available in the operating room and it is difficult to complete a modified Kessler suture with this straight needle. In our experience the plastic cannula with stylet is not strong enough and it is easily kinked in the tendon sheath making it difficult to be advanced to the distal palmar crease under the tendon sheath. Also the length of the cannula is hardly enough to reach the distal palmar crease and this technique is not suitable to use for tendons retracted to palm.

In summary there are three main advantages of this technique. First, the proximal tendon stump is not frayed due to undue multiple suture passages, only one permanent suture passage is enough for the retrieval of the proximal tendon stump in this technique. Second, there is no additional bulky mass at the end of proximal tendon stump, which may harm the tendon sheath during the passage and makes the passage difficult under the pulley system. There is only one permanent suture placed in the proximal tendon stump in this technique, which creates a negligible additional mass. Third, there is no need of special material, the catheter we used in this technique is readily available in the operating room. It has a smooth surface which minimally harms the tendon sheath during the passage. It is long enough to be used between the zone 2 and the distal palmar crease and also between the wrist and the distal palmar crease.

We have used this technique 21 times without any complication in our clinic. We have not seen any suture breakage during the passage or needle breakage due to the bending of the needle.

## Conclusions

We have found this technique is simple and very practical without need of any other material that found in operating room. This technique is also very effective in retrieving the retracted tendon stump without causing undue damage to the tendon stump or tendon sheath.

## Competing interests

The authors declare that they have no competing of interests.

## Authors’ contributions

MBÖ carried out the surgical procedures and drafted the manuscript. MK and TK participated in coordination of the study. SOB participated in the design of the study. MA conceived of the study, and participated in its design and coordination and helped to draft the manuscript. All authors read and approved the final manuscript.
